# Evaluation of the Microbiological Effectiveness of Three Accessible Mask Decontamination Methods and Their Impact on Filtration, Air Permeability and Physicochemical Properties

**DOI:** 10.3390/ijerph19116567

**Published:** 2022-05-27

**Authors:** Roberta Lordelo, José Rafael S. Botelho, Paula V. Morais, Hermínio C. de Sousa, Rita Branco, Ana M. A. Dias, Marco S. Reis

**Affiliations:** 1 Centre for Mechanical Engineering, Materials and Processes (CEMMPRE), Department of Life Sciences, University of Coimbra, 3000-456 Coimbra, Portugal; roblordelo@gmail.com (R.L.); rsrbranco@gmail.com (R.B.); 2 Chemical Process Engineering and Forest Products Research Centre (CIEPQPF), Department of Chemical Engineering, University of Coimbra, Rua Sílvio Lima, Pólo II—Pinhal de Marrocos, 3030-790 Coimbra, Portugal; rafaelciencia@gmail.com (J.R.S.B.); hsousa@eq.uc.pt (H.C.d.S.); adias@eq.uc.pt (A.M.A.D.)

**Keywords:** respiratory protective devices (RPD), decontamination methods, nebulized hydrogen peroxide, commercial bleach, microwave steam-sanitizing bag, microbiological effectiveness, filtration efficiency, air permeability, physicochemical properties

## Abstract

The need to secure public health and mitigate the environmental impact associated with the massified use of respiratory protective devices (RPD) has been raising awareness for the safe reuse of decontaminated masks by individuals and organizations. Among the decontamination treatments proposed, in this work, three methods with the potential to be adopted by households and organizations of different sizes were analysed: contact with nebulized hydrogen peroxide (H_2_O_2_); immersion in commercial bleach (NaClO) (sodium hypochlorite, 0.1% p/v); and contact with steam in microwave steam-sanitizing bags (steam bag). Their decontamination effectiveness was assessed using reference microorganisms following international standards (issued by ISO and FDA). Furthermore, the impact on filtration efficiency, air permeability and several physicochemical and structural characteristics of the masks, were evaluated for untreated masks and after 1, 5 and 10 cycles of treatment. Three types of RPD were analysed: surgical, KN95, and cloth masks. Results demonstrated that the H_2_O_2_ protocol sterilized KN95 and surgical masks (reduction of >6 log10 CFUs) and disinfected cloth masks (reduction of >3 log10 CFUs). The NaClO protocol sterilized surgical masks, and disinfected KN95 and cloth masks. Steam bags sterilized KN95 and disinfected surgical and cloth masks. No relevant impact was observed on filtration efficiency.

## 1. Introduction

Respiratory protective devices (RPD) are playing a major role in the control and mitigation of the COVID-19 pandemic. During outbreaks, their use was either obligatory or highly recommended, both in professional and social contexts. However, the massification in the use of RPD led to two main problems: (i) local shortages, especially at the beginning of the first outbreak and until producers reconfigure their throughput and adapt the logistic supply chains; (ii) the mass disposal of masks, which are plastic fibre-based products, is creating an environmental problem, as they are being collected and processed as undifferentiated domestic waste. The first problem, even though circumstantial and limited in time, may have a significant impact on public health and on critical operations. Although increasing production may solve this problem, it certainly does not mitigate the second one. A solution to both aspects consists of reusing RPD, under certain conditions and after an adequate decontamination treatment. This line of action could indeed address local shortages, as single reuse has the equivalent effect of increasing the availability of masks by 100%. It will also reduce the environmental footprint by 50% (again, just for single reuse). These arguments motivate the research of efficient and effective decontamination methods for RPD as well as the analysis of situations where they could be considered viable.

Two waves of research on the application of decontamination methods for the reuse of RPD can be found in the literature. The first occurred around 2010, following the Influenza A (H1N1) outbreak, and the second, starting in 2020, was pushed by the SARS-CoV-2 pandemic. During the influenza pandemic, a study reported the impact of five decontamination methods on the KN95, surgical and P100 masks’ integrity and performance, evaluating aerosol penetration and airflow resistance, among other aspects [1]. The authors pointed out UV irradiation, ethylene oxide and vaporized hydrogen peroxide as the most promising decontamination methods. On the other hand, the use of bleach raised some concerns due to a noticeable scent remaining after overnight drying and the presence of low levels of chlorine found in off-gassing experiments. Microwave irradiation was also not recommended due to the risk of melting of mask’s layers. Other work tested the use of three cycles of treatment for eight decontamination methods, including some of the above-mentioned procedures [2]. As before, filter aerosol penetration and filter airflow resistance were analysed together with several aspects of the RPD integrity, but the filtration efficiency or the ability to inactivate infectious biological organisms was not assessed. The effects of three decontamination methods, ultraviolet irradiation, moist heat incubation and microwave-generated steam, on the fitting characteristics, odour, comfort and or donning ease of RPDs were also explored [3]. Moreover, the ability of three energetic methods, microwave-generated steam, warm moist heat, and ultraviolet irradiation to decontaminate the H1N1 influenza virus was also evaluated [4]. All these methods led to >4-log10 reduction of the viable H1N1 virus, but the authors used an aerosol contamination method instead of the most common spiking procedure, similar to the procedure followed by Fisher and collaborators [5]. The use of microwave steam bags was also assessed as a methodology for inactivating MS2 bacteriophages on RPD following three cycles of contamination-decontamination [6].

With the coronavirus outbreak, the interest in decontamination methods for the safe reuse of RPD was renewed, and several review papers were published. A recent report presented a thorough overview of the use of masks, their engineering, life cycle and decontamination [7]. Moreover, several recent reviews focused on decontamination interventions for surgical masks [8,9]. In a review of the literature on the persistence of coronavirus on inanimate surfaces and the use of biocidal strategies for chemical decontamination, chemical inactivation of the coronavirus by sodium hypochlorite (commercial use) at a concentration of 0.1% (p/v) has been suggested to be effective in 1 min [10]. This study recommends a 1:50 dilution of the standard bleach formulation for use during a pandemic crisis.

The effect of decontamination by UV irradiation, vaporized hydrogen peroxide and dry heat in surgical and KN95 masks was also evaluated using pig coronavirus [11]. The results showed a reduction of more than 3 orders of magnitude of the indicator (porcine respiratory coronavirus, PRCV) in contaminated masks and therefore the authors considered the protocol effective and reproducible. A KN95 mask decontamination protocol based on microwaves and using easily accessible materials was recently developed [12]. In this work, 10 µL of the MS2 phage at the concentration 109 PFU mL^−1^ were inoculated on the KN95 masks and after exposure to steam in a microwave for 3 min, the viral load was reduced by 5 to 6-log10 in all trials, confirming the effectiveness of the decontamination method.

The research conducted in the 2010s contributed new and valuable information about the feasibility of using several decontamination methods for RPD reuse. However, some of the most successful solutions advocated, require expensive equipment and well-trained personnel. This is the case of using ethylene oxide, for instance, which may also raise concerns about the subsequent reuse of the masks due to the toxicity of the decontamination agent. Additionally, most studies published focused on the impact of the treatments on the filtering performance of the RPD and, with a few exceptions, do not access simultaneously the microbiological inactivation efficacy.

In the present work, we resume the research line of study concerning the effectiveness of decontamination methods for reducing the pressure on the demand of RPD and mitigating the harmful environmental impact of their excessive use and concomitant disposal. This study was designed by considering the following criteria, selected with the premise to produce knowledge that help decision-makers to structure an effective response to the aforementioned health and environmental threats: (i) decontamination methods should be affordable and the protocols technically accessible to personal from small and medium enterprises (SME), small health services, nursing homes, public services (police, firefighters, etc.), households and alike; (ii) contemplate the most common masks used for professional and non-professional use; (iii) the assessment contemplates both microbiological decontamination efficacy and physical-chemical aspects.

Altogether, these criteria aim at achieving a leveraging effect on a large portion of society, encompassing both professional and non-professional contexts. Moreover, they address the needs of users who have different risk profiles and therefore use masks with distinct protective requirements. Finally, there is also the aim of increasing the current state-of-the-art on mask decontamination methods, by providing a comprehensive picture of the effects of using several treatment cycles.

According to these criteria, this study contemplated three types of RPD, surgical, KN95 and cloth masks and three decontamination procedures: contact with nebulized hydrogen peroxide (H_2_O_2_); immersion in commercial bleach (NaClO) (sodium hypochlorite, 0.1% p/v); and contact with steam in microwave steam-sanitizing bags (steam bag).

## 2. Materials and Methods

### 2.1. Respiratory Protective Devices (RPD)

**Figure 1:**
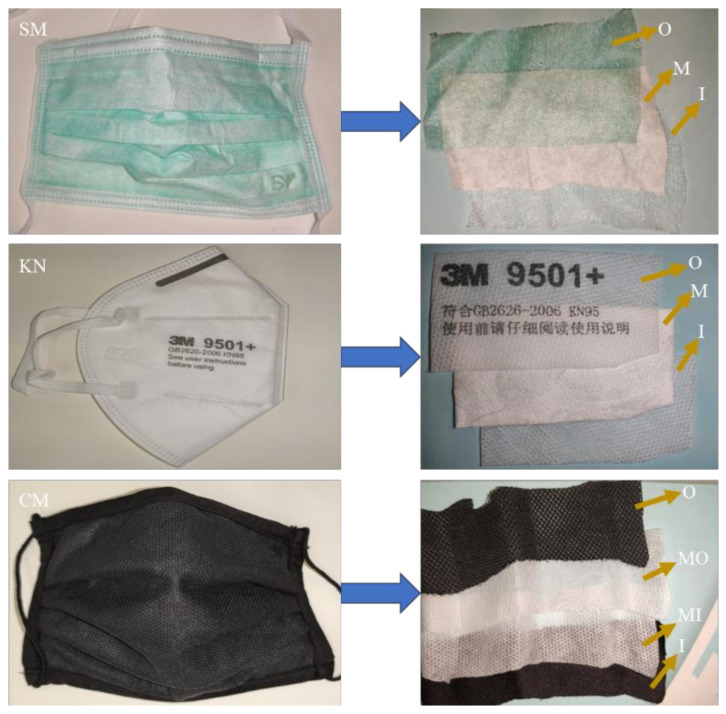
Images of the studied RPD. Top: surgical masks (**SM**); Middle: KN95 masks (**KN**); and Bottom: cloth masks (**CM**). RPD constituent layers: outer layer (O), inner layer (I), and intermediate layer (M). For CM-type masks, the existing intermediate layers were indicated as intermediate layers close to the outer layer (MO) and as intermediate layer close to the inner layer (MI).

Three types of RPD were studied in this work: surgical masks (SM), KN95 masks (KN) and cloth masks (CM). The surgical masks with ties were acquired from the producer, Bastos Viegas (Ref Number 465-001, Bastos Viegas, S.A., Penafiel, Portugal). Cloth masks with elastic bands on the side were manufactured by Borgstena (Concept 2 B, Borgstena Textile Portugal, Nelas, Portugal). The KN95 masks tested were from the model GB2626-2006 9501+ KN95 of the 3M brand. Figure 1 shows details of the RPD used and their different constituent protective layers. According to the information provided by the manufacturers, all 3 types of masks comprise different poly(propylene) (PP) and poly(ethylene terephthalate) (PET) layer materials, as follows:

Surgical masks (SM): outer layer (O, non-woven spun-bonded PP); intermediate layer (M, non-woven melt-blown PP); and inner layer (I, non-woven spun-bonded PP)

KN95 masks (KN): outer layer (O, non-woven spun-bonded PP); intermediate layer (M, non-woven melt-blown PP “cotton”, with treatment for achieving the desired electric properties); and inner layer (I, non-woven spun-bonded PP)

Cloth masks (CM): outer layer (O, PET woven fabric); intermediate layer closer to the outer layer (MO, non-woven spun-bonded PP); intermediate layer closer to the inner layer (MI, non-woven spun-bonded PP); and inner layer (I, PET woven fabric—different from the outer layer)

### 2.2. Decontamination Treatments

Three distinct decontamination methodologies were performed for all studied RPD, namely: (i) contact with nebulized hydrogen peroxide (H_2_O_2_); (ii) immersion in commercial bleach (NaClO) (i.e., sodium hypochlorite 0.1% p/v); and (iii) contact with steam in microwave steam-sanitizing bags (steam bag). All studied RPD were submitted to 5 and 10 consecutive treatment cycles (designated as 5c, 10c, respectively; for the microbiological assays, 1 cycle was also considered, referred to as 1c), according to the procedures detailed below. Control groups (RPD without treatment) were also contemplated, to establish the baseline for comparison.

#### 2.2.1. Contact with Nebulized Hydrogen Peroxide (H_2_O_2_)

RPD were contacted with nebulized hydrogen peroxide in a sealed room (10 m^3^), at room temperature (18 ± 2 °C). Hydrogen peroxide vapours were generated by a commercial nebulizer (NOCOLYSE^®^, model Nocospray, Oxypharm, Champigny-sur-Marne, France), which sprayed liquid hydrogen peroxide into the sealed room until achieving a concentration of 3 mL of nebulized liquid per m^3^, according to the manufacturer protocol. More specifically, each treatment consisted of the emission of hydrogen peroxide in two applications with an interval of 30 min between the first and the second application, and an interval of 2 h 30 min after the second application and the next new treatment (manufacturer’s recommendation).

After the contact period, RPD were removed from the room and were kept for 3 h in order to allow for the evaporation of residual hydrogen peroxide adsorbed/absorbed in the RPD. The procedure was repeated in order to obtain RPD processed for 1, 5 and 10 consecutive treatment cycles. In the end, RPD were stored in sealed food-grade poly(ethylene) (PE) plastic bags for further characterization. Experiments were carried out in quintuplicate for physicochemical characterization.

RPD undergoing microbiological assays were inoculated prior to the 1st, 5th and 10th treatment cycles, with a suspension of endospores of *Geobacillus stearothermophilus* DSM22. Inoculation was performed by applying 100 µL of this suspension on two different parts of the RPD, according to ISO 14937 [13] and dried, at room temperature, in a laminar flow chamber, before performing the microbiological analysis (see below).

#### 2.2.2. Immersion in Commercial Bleach (NaClO)

The bleach disinfection for physicochemical characterization followed the protocol of Kampf and collaborators [10]. Briefly, RPD were immersed in bleach aqueous solutions (0.1% p/v of sodium hypochlorite) diluted with deionized water from a 3% p/v commercially available bottled bleach (Lixívia A Limpinha, Elismarc, Salreu, Portugal), only used for 3 straight days after being opened. Immersion was carried out for 1 min at room temperature (18 ± 2 °C) followed by rinsing with distilled water 10 times and dried in a forced air-circulating oven at 30 °C for 4 h, for physicochemical characterization, or at room temperature at 18 ± 2 °C for 48 h, for microbiological assays. All the described procedures were repeated to obtain RPD processed with bleach for 5 and 10 consecutive treatment cycles. Experiments were carried out in quintuplicate. Finally, RPD were stored in sealed food-grade poly(ethylene) (PE) plastic bags for further characterization experiments.

RPD for microbiological assays were inoculated prior to the 1st, 5th and 10th treatment cycles, with a suspension of endospores of *Bacillus atrophaeus* DSM675, as described in Section 2.2.1.

#### 2.2.3. Contact with Steam in Microwave Steam-Sanitizing Bags (Steam Bags)

RPD for physicochemical characterization were treated as described in the literature [6], with small adjustments. Briefly, RPD without the metallic parts and 60 mL of deionized water (according to supplier instructions) were laid inside commercially available steam-sanitizing plastic bags (Quick CleanTM microwave bag, produced by Medela^®^, Barcelona, Spain), and microwaved for 3 min at 800 W and 2450 MHz (MS23K3513AW, Samsung, Portugal). After processing, RPD were removed from steam bags and air-dried at room temperature. The same procedure was repeated to obtain RPD processed for 5 and 10 consecutive microwave treatment cycles. Experiments were carried out in quintuplicate, and RDP were stored in sealed food-grade poly(ethylene) (PE) plastic bags for subsequent characterization. For microbiological assays, RPD were treated as for the H_2_O_2_treatment, using endospores of *G. stearothermophilus*.

### 2.3. Microbiological Assays

#### 2.3.1. Endospores Production

The present study followed the standard guidelines ISO 14937 [13] and the FDA Biological Indicators Guide (Food and Drug Administration) for *G. stearothermophilus* and *B. atrophaeus* endospores production. Briefly, *G. stearothermophilus* cells were inoculated in Tryptone Soy Broth (TSB), incubated at 55 °C for 72 h and 160 rpm [14]. *B. atrophaeus* cells were grown in Nutrient Broth (NB) medium at 30 °C for the same period of time and rotation level. After bacterial growth, cells were centrifuged at 4 °C and pasteurized at 80 °C for 10 min; adapted from Wells–Bennik et al. (2019) [15]. To release the endospores, the cells were lysed under high pressure (1500–2000 psi) [16], and the endospores were concentrated by centrifugation using a 100 kDa centrifugal filter at 4000 rpm for 30 min. The endospore suspension quality was evaluated by microscopy visualization and kept at 4 °C.

The concentration of endospores in the suspensions was determined by plating serial dilutions on a solid medium. After incubation at the respective temperatures, the colony-forming units (CFU) were enumerated. Each suspension was diluted to obtain stock suspensions with approximately 107, 108 and 109 cells mL^−1^, and used as the microbial concentrations for the masks decontamination studies. The inoculums were placed in different positions of the masks and marked for later collection of the sample after the treatment sequence was completed.

#### 2.3.2. Mask Contamination Protocol

RPD decontamination was evaluated, at least in triplicate, after a given sequence of treatment cycles—A treatment cycle corresponds to the complete execution of a given decontamination method until the mask is dried and ready for reuse. The number of treatment cycles was 1, 5 and 10 cycles. In order to proceed with the microbiological analysis, the RPD corresponding to the 5c and 10c groups were removed at the end of the fourth and ninth cycles, respectively. The RPD (for the treatments and controls) were inoculated with 100 µL of each stock suspension [13,17], dried overnight at room temperature, and submitted to treatment. The control groups were contaminated, dried overnight at room temperature, but not subjected to treatment in order to make a comparative analysis.

#### 2.3.3. Microbiological Analysis

After undergoing the target number of treatment cycles, the masks were subjected to microbiological analysis. For such, the inoculated area of both masks undergoing treatment and the controls was removed and suspended in 0.85% saline solution for 1 h. Then, 100 µL of this solution was plated on Nutrient Agar (NA), or Tryptic Soy Agar (TSA) medium, and incubated for 24 h at 30 °C or 50 °C, according to the microorganism analysed. Following the ISO 14937 Standard [13] and to assess the efficiency of the disinfection method, the CFUs were counted.

### 2.4. Filtration Efficiency and Air Permeability of RPD

For all tests, three masks were analysed in two positions each, conducting 6 experimental points for each experimental condition under analysis (combination of mask type and treatment applied).

#### 2.4.1. Filtration Efficiency

Measurements of particle filtration efficiency (PFE) were performed according to the standard CWA 17553:2020 Annex D. Particle concentrations upstream (before passing through the filter medium) and downstream (after passing through the filter medium) were determined with resort to a laser diode (spherical). Solid unneutralised particles of PSL (polystyrene latex) were suspended in the air at a concentration of 40 ± 20 particles cm^−3^. The flow of air was set to 28.3 L·min^−1^ with a testing time of 1 min. The testing area of the sample was 80 ± 12 cm^2^. The filtration speed was set to 6 ± 1 cm·s^−1^. The PFE measurements are obtained from the percentage penetration, according to Equations (1) and (2):

PFE(%) = 100% − Penetration (%)
(1)

Penetration (%) = (Particle concentration downstream)/(Particle concentration upstream) × 100%(2)

In the equations above, the upstream and downstream concentrations are relative to particles with a diameter of approximately 3 ± 0.5 µm.

#### 2.4.2. Air Permeability

The measurements of air permeability were performed according to the standard ISO 9237:1995. The pressure drop applied was set to 40 Pa. The testing area of the sample was 20 cm^2^. Ambient conditions were maintained at 20 ± 2 °C and 65 ± 4 % of relative humidity (RH). Measurements of the air permeability are provided in units of l m^−2^·s^−1^ or mm·s^−1^.

### 2.5. Physicochemical Characterization of RPD

The surfaces of all the constituent layers of the RPD (both for non-treated and for treated samples using all methods for 5 and 10 treatment cycles) were analysed. All samples were analysed in duplicate (at least).

#### 2.5.1. Fourier Transform Infrared Spectroscopy Coupled with Attenuated Total Reflection (FTIR-ATR) Analysis

Chemical composition was studied by FTIR-ATR using a diamond lens (Frontier, Perkin Elmer, Waltham, MA, USA). Samples from all RPD layers were analysed at constant force (65 N) and scanned (64 scans) with a 4 cm^−1^ resolution. Analyses followed a well-established method for processed polymeric materials containing additives [18]. Results were analysed using dedicated software (Spectrum 10).

#### 2.5.2. Contact angle Measurements

Samples (rectangular shape, 6 × 4 cm^2^) were analysed for water contact angles (OCA 20, Dataphysics Instruments GmbH, Filderstadt, Germany), following a well-established sessile drop method [19]. Static water contact angles were measured at room temperature (18 ± 2 °C) using 10 µL droplets of distilled water. Contact angle values were calculated by the Laplace-Young method (software SCA 20).

#### 2.5.3. Water Vapor Transmission Rates

The rates of water vapor transmission (WVTR) were determined according to E96-90 ASTM International Standard (inverted cup, method D), with some modifications. Circular samples (1 cm diameter) obtained from integral RPD (all layers) were placed as a permeation membrane between a 95% RH atmosphere (generated by a saturated potassium sulphate aqueous solution) and a dried silica gel sample contained in a glass vial (approx. 2 g, saturation before 100 h of testing was avoided). Water vapor permeation measurements were carried out considering two flux directions: (i) from the outer to the inner layer of RPD to simulate flux from the atmosphere to the user (i.e., potential environmental contamination to the user); and (ii) from the inner to the outer layer of RPD to simulate flux from the user to the atmosphere (i.e., user breathing/coughing). The mass of water vapor permeated through each RPD (in both flux directions) was measured and correlated up to 24 h. WVTR values were calculated according to the following equation:

WVTR = m/(A × ∆t)(3)

where m (g) is the mass of water vapor permeated during the time period Δt (h) and A (cm^2^) stands for the effective transfer area.

#### 2.5.4. Thermogravimetric Analysis (TGA)

Analyses were performed on samples (approx. 8 mg) of RPD using a thermogravimetric analyser (Q500, TA Instruments, Grimsby, ON, Canada). Samples were heated from 25 °C to 600 °C (at 10 °C/min) under a dry nitrogen inert atmosphere (100 mL·min^−1^). Degradation temperatures were defined as the extrapolated onset temperatures, according to the ISO 11358-1 standard. Results were treated using instrument software (Universal Analysis 2000, TA Instruments).

#### 2.5.5. Modulated Differential Scanning Calorimetry (MDSC)

All the constituent layers of the RPD were analysed for their thermal phenomena (melting, and crystallization temperatures) using a differential scanning calorimeter (Q100, TA Instruments, Canada) in modulated mode (+/− 0.64 °C every 60 s). Samples (approx. 8 mg) were hermetically sealed in alumina pans and: (i) heated from −80 °C to 200 °C at 10 °C/min (for PP-based layers); (ii) heated from 0 °C to 350 °C, cooled again up to 0 °C and re-heated up to 350 °C always at 10 °C/min rate (for PET-based layers). All samples were analysed under dry a nitrogen atmosphere (50 mL·min^−1^). Results were treated using adequate software (Universal Analysis 2000, TA Instruments).

#### 2.5.6. Mercury Intrusion Porosimetry (MIP)

Average porosity and average pore size values of RPD (all layers) were determined by mercury intrusion porosimetry (Autopore IV 9500, Micromeritics, Norcross, GA, USA). The experiments were performed according to the methodology defined in ISO 15901-1:2016).

#### 2.5.7. Optical and Electronic Microscopy

All the constituent layers of studied RPD (for non-treated samples) were preliminarily analysed by optical microscopy, using a digital microscope (X000PGOALR, Jiusion, 800×, China). Afterwards, all RPD (all layers) were analysed by Field Emission Gun Scanning Electron Microscopy (FEG-SEM) (Merlin, Carl Zeiss, Oberkochen, Germany). RPD samples were sputter-coated with gold for 15 s (around 4 nm thickness) and analysed (at 2 kV) at several magnifications (mostly 62× to 2500×).

### 2.6. Data Analysis and Statistics

The software JMP-PRO version 15.0 (from SAS Institute, Inc., Cary, NC, USA) was used to visualize and perform statistical analysis. Differences between treatment cycles for each methodology were assessed using a non-parametric test. The two-tailed Kruskal–Wallis test based on ranks was applied for the experiments regarding filtration efficiency, air permeability and physicochemical characterization. As for the analysis of microbiological assays, the statistical analyses of the differences between the treated and untreated inoculated masks groups were performed using GraphPad Prism 6 (Graph-Pad Software, San Diego, CA, USA). The comparison between different treatment cycles of the same type of masks was also conducted with this software. *p*-values were calculated using an independent sample *t*-test (to compare each treatment of interest with the control), where the following notation was adopted: **, ***, **** mean a statistically significant difference from the control, at significance levels of 0.01, 0.001 and 0.0001, respectively; “ns” stands for a non-significant difference at a significance level of 0.05 (*p*-value > 0.05).

## 3. Results and Discussion

### 3.1. Assessment of the Microbiological Effectiveness

**Figure 2:**
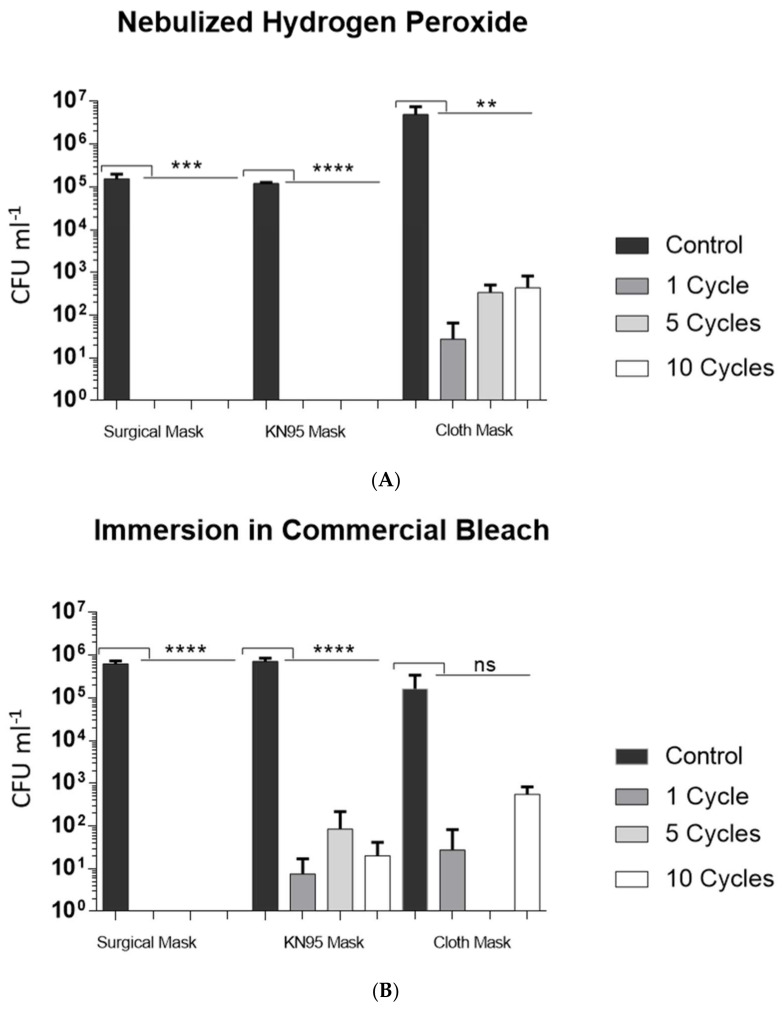

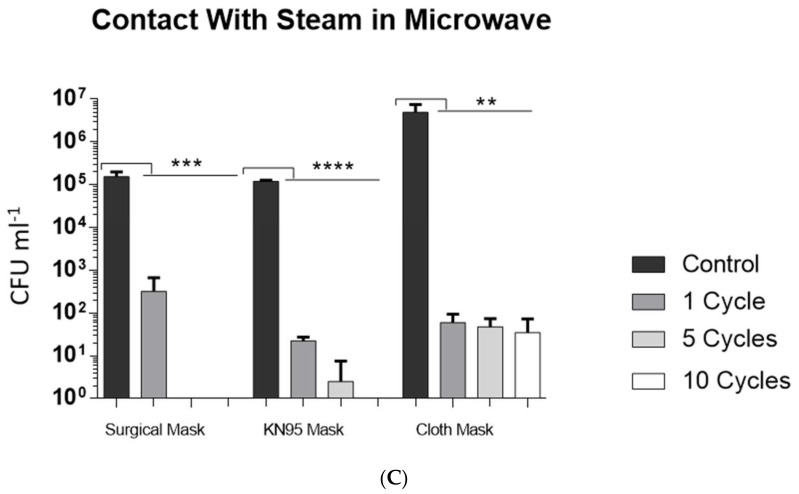
Decontamination of three types of RPD using different procedures: nebulized hydrogen peroxide (**A**), immersion in commercial bleach (**B**) and contact with microwave-generated steam (**C**). Surgical, KN95 and cloth masks were inoculated with indicator endospore suspension with approximately 107 endospores mL^−1^. After the treatment cycles (1c, 5c and 10c), the endospores survival was evaluated by plating assay. Untreated RPD were included as controls in all assays. Data shown are the mean values (±standard deviation) obtained from three or four replicates. The type of mask (surgical, KN95 and cloth masks) is presented in the X-axis of the three plots. Ns—not significantly different, **, ***, **** signal a statistically significant difference from the control, at significance levels of 0.01, 0.001 and 0.0001, respectively.

All decontamination procedures resulted in a reduction of CFUs when compared to the control assays, for all the three levels of treatment cycles (1c, 5c, 10c) (Figure 2). Nebulized hydrogen peroxide reduced 4 to 5 orders of magnitude the microbiological indicator inoculated in the cloth masks, and more than 6 orders of magnitude in surgical and KN95 masks after the treatment cycles. Therefore, nebulized hydrogen peroxide treatment effectively resulted in a significant reduction of CFUs recovered when compared to the CFUs obtained from untreated masks (control), for all types of RPD, (Figure 2A).

Decontamination by immersion in commercial bleach reduced 7 and 5 to 6 orders of magnitude of total endospores inoculated in surgical and KN95 masks, respectively. The statistical analysis of the results showed high significance (*p*-value < 0.0001) for both surgical and KN95 masks. The treatment cycles performed in cloth masks did not show a statistically significant reduction of CFUs recovered (*p*-value of 0.1188) comparatively to the baseline controls (Figure 2B).

The treatment by contact with microwave-generated steam reduced the CFUs of KN95 and cloth masks by 5 orders of magnitude, overall. In turn, this procedure has reduced CFUs in surgical masks by 4 orders of magnitude for 1c, and 6 orders of magnitude for 5c and 10c (Figure 2C).

The inactivation process with nebulized hydrogen peroxide was 100% efficient in KN95 masks, leading to a reduction of 6 orders of magnitude, but showed lower efficiency for cloth masks decontamination. Inactivation with commercial bleach resulted in a reduction of more than 6 orders of magnitude for all surgical masks but has an efficiency of 78% and 67% for the KN95 and cloth masks analysed, respectively. Microwave steam inactivation was shown to be a very efficient decontamination process for all KN95 masks. However, in 66% of the surgical masks and in 55% of the cloth masks, the endospores inactivation was not complete.

In this work, it was not possible to relate the number of endospores inoculated with the higher or lower efficiency of a treatment since it varied with the RPD used.

The number of cycles of decontamination affected the efficiency of decontamination, often decreasing the efficiency of cloth masks. The decontamination efficiency of cloth masks varied according to the number of endospores inoculated, the number of cycles of disinfection and the technique used.

**Figure 3:**
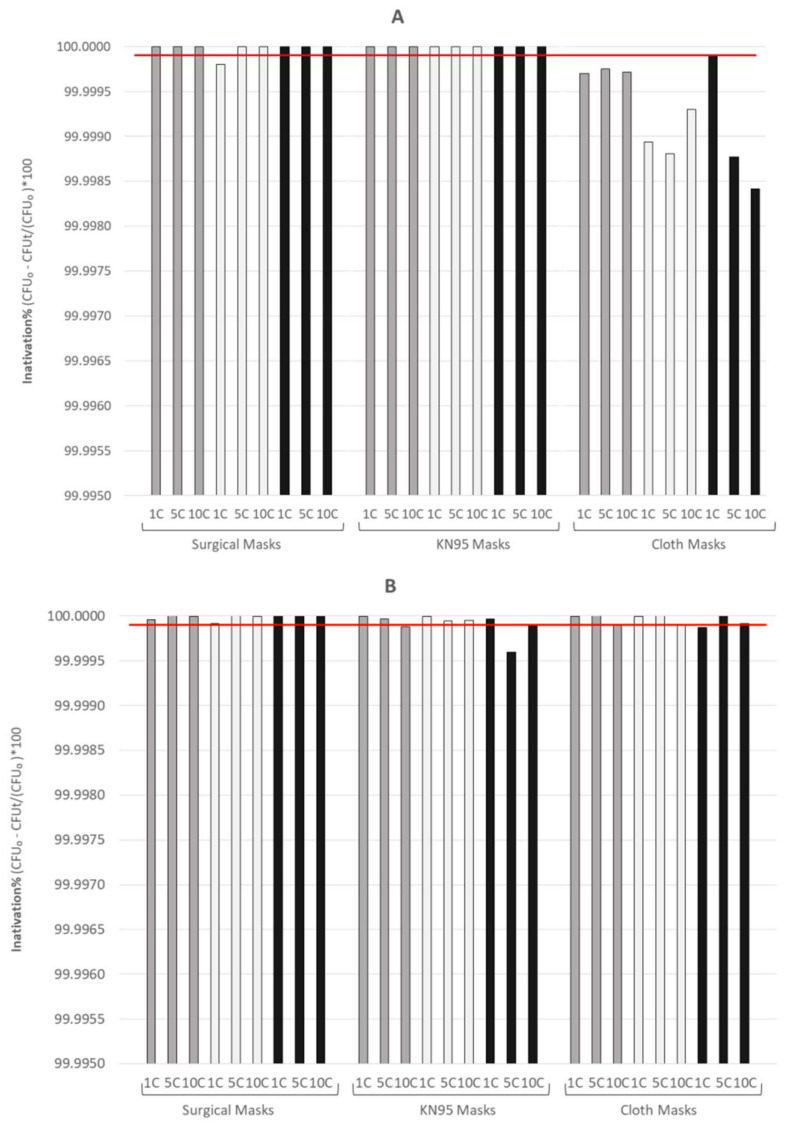

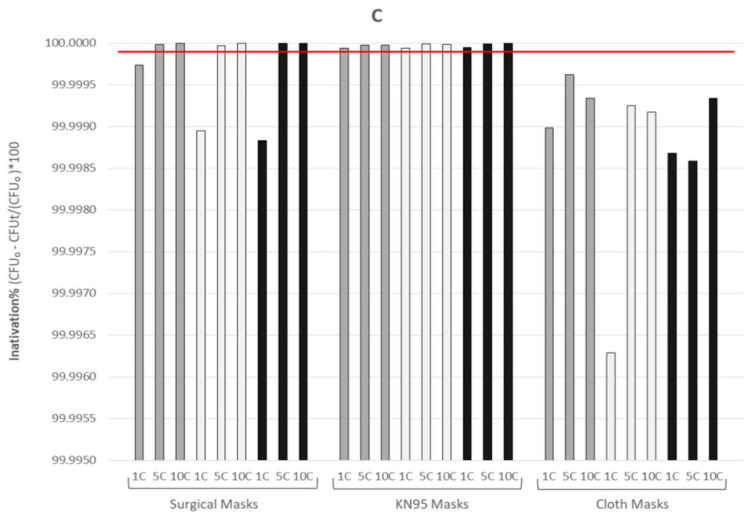
Effectiveness of endospores’ inactivation using three different endospores number suspensions (10^7^ (black bars), 10^8^ (grey bars), and 10^9^ (white bars)) by the three decontamination processes for surgical, KN95 and cloth masks: nebulized hydrogen peroxide (**A**), immersion in commercial bleach (**B**) and contact with microwave-generated steam (**C**). The red line indicates the Standard Effectiveness established in ISO 14937 (corresponding to a reduction of six orders of magnitude in the CFUs). The inactivation values were obtained through the ratio between the difference of the inoculated CFUs and the CFUs recovered after treatment and the total inoculated CFUs.

Treatments with nebulized hydrogen peroxide resulted in the highest reduction of CFUs recovered on the masks tested, presenting the highest decontamination effectiveness. Other studies also referred to this process as presenting several advantages in manipulation and use [8,11,20,21]. Surgical and KN95 masks showed total inactivation of the microbiological indicator, according to ISO 14937 standards (Figure 3A).

The second-best decontamination strategy in terms of microbiological decontamination effectiveness was the immersion in commercial bleach (Figure 3B), which led to endospores inactivation efficiency of 99.9999% and 99.9990% for surgical and KN95 masks, respectively (*p*-value < 0.0001). Despite the high effectiveness shown in these two types of masks, only surgical masks achieved the ISO standard’s target for sterilization. The statistical analysis of the treatments performed in cloth masks showed non-significance w.r.t. to the control, even though the overall decontamination efficacy of the protocol was 99.999%. However, the treatment by immersion in commercial bleach presents some limitations, such as the wide range of concentrations of sodium hypochlorite in commercial bleach available in the market, and the dependence on the time after the first utilization. These factors need to be taken into account to ensure an efficient and reproducible decontamination [21,22].

The statistical analysis performed on all RPD in contact with microwave-generated steam led to a significant reduction in the number of CFUs. The KN95 masks obtained a high degree of significance (*p*-value < 0.0001), and this result was confirmed by the analysis of the efficiency of all the treatment cycles. The process achieved a reduction of 6 orders of magnitude for KN95 masks, which corresponds to an inactivation efficiency of 99.9999%, meaning that it can be considered effective, following the criterion of Standard Effectiveness established in the ISO 14937:2000 standard (Figure 3C). The microwave-generated steam decontamination of surgical (except for 1c) and cloth masks showed a reduction between 4 and 5 orders of magnitude. Similar published works considered the capacity of the steam generated by the microwave to be effective, with a reduction in the number of microorganisms around 4–6 log [12,23].

### 3.2. Effects of Treatments on Filtration Efficiency

**Figure 4:**
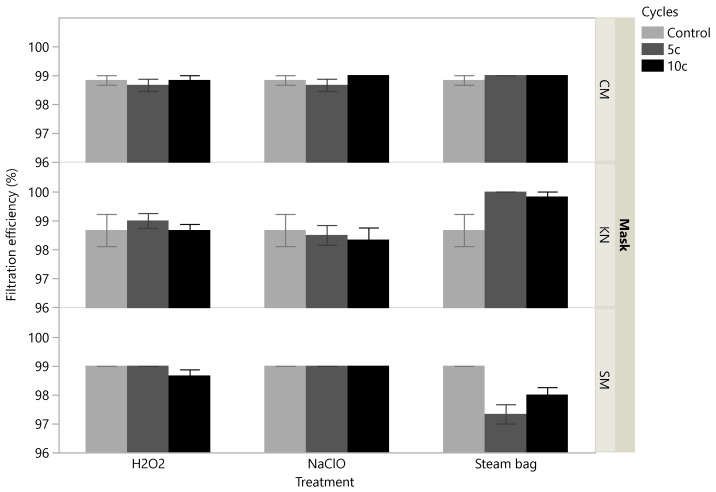
Experimental results obtained for the filtration efficiency, stratified according to the type of mask (CM, KN, SM) and decontamination treatment (H_2_O_2_, NaClO, Steam Bag). Each bar corresponds to the mean of all measurements obtained for each level of cycles (6 replicates); the error bars depict the standard error of the mean.

The impact of the different treatments on the masks’ filtration efficiency was minor for the cloth masks tested, which maintained a PFE close to 99% with small impacts arising from the number of decontamination cycles, which in most cases are not statistically significant (Figure 4, Table 1). The filtration efficiency of KN95 masks exhibited a higher variability across the cycles, but still maintained an average level of 98% efficiency or even above this baseline, as happened for several cycles of decontamination using steam bags, showing significant differences w.r.t. to the control (low p-value for the Kruskal–Wallis test—0.0067). The effects of treatment cycling using the H_2_O_2_ and NaClO were comparatively smaller and lacking in statistical significance (Table 1). The filtration efficiency of surgical masks was essentially unaffected by the number of cycles applied for the NaClO treatment and seemed to experience only a small and non-significant average reduction for higher number of cycles of H_2_O_2_ treatment (*p*-value = 0.1194). However, a statistically significant reduction in the efficiency of surgical masks could be observed when steam bags were used; the magnitude of the decay was in the range of 1–2% of efficiency loss, leading to PFE of the order of 97–98%.

### 3.3. Air Permeability Tests

**Figure 5:**
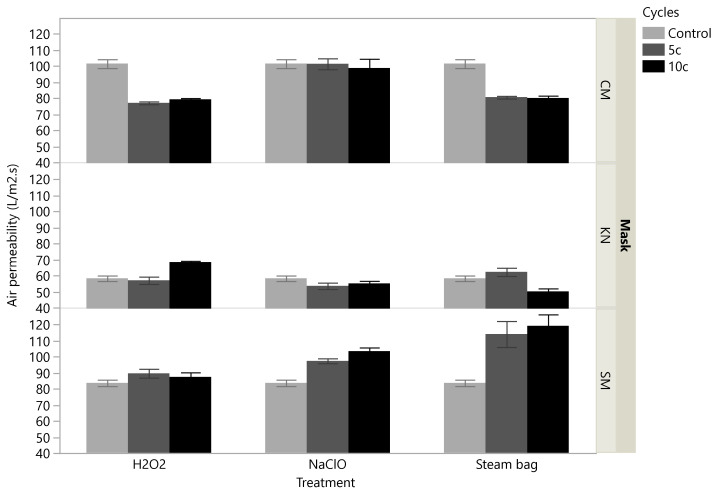
Experimental results obtained for the air permeability, stratified according to the type of mask (CM, KN, SM) and decontamination treatment (H_2_O_2_, NaClO, Steam Bag). Each bar corresponds to the mean of all measurements obtained for each level of cycles (6 replicates); the error bars depict the standard error of the mean.

The base levels of air permeability (for the control replicates) were quite different for the different types of masks (Figure 5). Cloth masks showed higher permeability, followed by surgical masks, while KN95 exhibited the lowest permeability level. The NaClO treatment exerted a low impact on the permeability of cloth and KN95 masks, without statistical significance (Table 2). Even though this treatment has led to a progressive increase in air permeability for surgical masks, their filtration efficiency was maintained (Figure 5 and Table 2). The air permeability after decontamination with H_2_O_2_ tended to decrease for all cloth masks but increased for the KN95 masks submitted to 5 treatment cycles. Surgical masks were the less affected by H_2_O_2_ treatments. Steam bags showed a significant impact on the air permeability of masks: increasing for surgical masks (as happened with the use of NaClO) and decreasing for cloth masks. KN95 masks were also affected, but only for a larger number of cycles of steam contact, decreasing their air permeability. In general, treatments tended to increase the air permeability of surgical masks (NaClO and steam bags), whereas, in the case of cloth masks, the air permeability tended to be reduced with the increase in the number of treatment cycles (H_2_O_2_ and steam bags). These observations may conjecturally be explained by a progressive deterioration of the matrix in the case of surgical masks, and progressive tissue collapsing in the case of cloth masks. The behaviour of KN95 masks was more erratic: an increase was registered for H_2_O_2_ treatment while a reduction was observed when steam bags were used.

### 3.4. Effects of Treatments on the RPD’s Physicochemical Properties and Structure

#### 3.4.1. FTIR-ATR Analysis

FTIR-ATR results (Figure S1, Table S1) confirmed that all tested RPD layers match the suppliers’ information regarding their corresponding chemical compositions. This was also confirmed by running our data on sample libraries/databases (e.g., HR Nicolet Sampler Library, Cross Sections Wizard, and Hummel Polymer Sample Library). The results showed that no chemical changes seem to have occurred on the surfaces of all the constituent layers of the studied RPD, as the result of the employed decontamination procedures and treatment cycles. Only very small variations were observed in the wave numbers of the peaks corresponding to the main chemical groups of PP and PET (Table S1), and considering the employed resolution of the FTIR-ATR (4 cm^−1^). Moreover, no additional absorbance bands appeared after the employed procedures and treatment cycles, which eventually may be due to any new substances that were produced by potential chemical degradation reactions.

#### 3.4.2. Contact Angle Measurements

The surface hydrophobicity of RPD layer materials plays a critical role on the ability of blocking liquid droplets containing airborne microorganisms, especially on their outer layers [24,25,26]. The measured static water contact angles are presented in Table S2, where it could be observed that all contact angles measured for the distinct layers of all non-processed (control) masks were well above 90° (between 115.4° and 151.3°, for PP-based layers, and 132.9°, for the only PET-based layer). This indicates as expected that all the layer materials employed in these RPD originally presented a high hydrophobic nature [25,27,28,29].

A comparison with control/non-processed RPD, showed that the three employed decontamination methodologies, for 10 treatment cycles, led to some changes in the measured water contact angle values. In general terms, these variations were typically an increase of the contact angle values for the distinct layers of SM and CM (all made of PP and PET), which is typically a good feature, and a decrease of these values for the distinct layers of KN (all made of PP). However, the statistical analysis indicated that only the changes observed for the distinct layers of KN95 masks (as a result of all employed decontamination treatments) and the changes promoted by the steam bag method (for all layers of all tested RPD) could be considered statistically significant (*p*-value < 0.05). Therefore, we could conclude that the contact angle (and thus the wettability) of the distinct layer materials of KN95 masks seemed to be more prone to be affected by all the employed decontamination treatment processes (namely at their inner and intermediate layers), and these effects seemed to be more pronounced for the H_2_O_2_ and the NaClO treatments. On the other hand, amongst all employed treatments, the steam bag method seems to be the treatment that leads to more statistically significant variations in the contact angle values (*p*-value < 0.05), namely for all the KN and CM constituent layer materials. Since FTIR-ATR results did not show any clear chemical modification/degradation of constituent layer materials, the observed changes in contact angle values may be due to some unclear physical and/or morphological surface changes induced in layer materials by the processing methods (e.g., nano- and microfiber leaching, water/vapour sorption/desorption, drying procedures, variation of surface electrostatic charges, temperature variation, extraction of specific processing additives—e.g., repellents, plasticizers), by sample manipulation (e.g., forces/stresses during sample handling which may affect sample morphology), or by the sampling preparation process (e.g., RPD layer materials heterogeneity—in terms of fibre density, porosity, roughness and organization) [30,31,32,33].

#### 3.4.3. Water Vapour Transmission Rate (WVTR)

In addition to safety and to biological concerns, the water vapour transmission rates (WVTR), together with the pressure drop across RPD, and the temperature and relative humidity inside the masks, are important aspects of the breathing and comfort-related functional properties of these types of biomedical devices [26,34,35,36].

WVTR results are presented in Figure S2 and Table S3. As happened in the case of the contact angle results, after 10 treatment cycles some variations in the WVTR values for SM and KN masks, all decontamination treatments maintained or slightly increased the WVTR (for both flux directions). In addition, the NaClO treatment seemed to be affecting to a higher extent the SM masks, increasing their WVTR. The H_2_O_2_ and the steam bag treatments affected KN masks to a higher extent, slightly increasing the WVTR, in both flux directions; for steam bag treatment, these treatments decreased the WVTR in the case of flux from the user to the atmosphere. Finally, for CM masks, it was observed that the H_2_O_2_ and NaClO treatments decreased the WVTR values (for both flux directions), while the steam bag treatment slightly decreased or increased the WVTR for flux from the atmosphere to the user or flux from the user to the atmosphere, respectively. However, from the statistical analysis (Table S3) of the WVTR results, these trends were not found to be statistically significant (all *p*-values > 0.05), which may be due to the low number of duplicates used.

#### 3.4.4. Thermogravimetric Analysis (TGA)

Thermogravimetric analyses (TGA) of the distinct layers of non-processed (control) RPD were carried out mostly to detect any potential chemical or physical differences between the distinct PP and PET layers, as well as to determine the analytical conditions to be later employed on Modulated Differential scanning calorimetry (MDSC) tests (see Table S4). TGA results showed that thermal degradation temperatures occur between 437.4 °C and 438.6 °C (for the non-woven spun-bonded PP layers, in SM, KN and CM masks), 438.6 °C (for the non-woven melt-blown PP layer, in SM masks), 372 °C (for the non-woven melt-blown PP “cotton”, with treatment for electret properties, in KN masks), and 403.2 °C (for both PET woven fabric layers, in CM masks), which are in the typical ranges found in the literature for these types of materials/products [37,38,39,40,41,42,43]. A similar agreement with the literature was also observed for the ashes/residual masses obtained at 595 °C for the three types of RPD layer materials. It was noticed that PET-based layers left much higher amounts of ashes/residues than PP-based layers (15–16%), which was certainly due to the different thermal oxidation reactions that were involved in each case, or to the presence of inorganic-based processing additives (e.g., pigments and flame retardants—which are quite usual at PET-based fabrics). Finally, the internal PP layer of KN masks degraded at much lower temperatures (372 °C) and left less ash/residues than all the other PP-based layers present in studied RPD. This was probably due to different PP molecular weights and to a higher isotacticity degree of this intermediate melt-blown PP layer, which is known to affect radical-based reactions in the thermal oxidation degradation of PP leading to higher thermal stabilities than in the case of less isotactic PP [42].

#### 3.4.5. Modulated Differential Scanning Calorimetry (MDSC)

The permanent or temporary contact of RPD layer materials with chemicals, water and water vapor, alone or together with temperature, as it occurs when applying the studied decontamination treatments, may affect some of the involved polymeric materials’ thermomechanical properties (e.g., by sorption and by reactions of chemicals and water with fibres, by extraction/lixiviation of processing/functional additives, or simply by the induction of thermal-based modifications in polymeric chain organization and crystallization properties). MDSC was employed to obtain results concerning the melting and crystallization temperatures of the distinct layers of non-processed (control) and processed (10 cycles) SM, KN and CM masks (Table S5).

MDSC results showed that, after 10 treatment cycles for the H_2_O_2_, NaClO and steam bag treatments, only no statistically significant variations were observed for the melting temperature values of the original PP-based layers. The values are in the typical ranges found in the literature for these types of PP- and PET-based materials/products [44,45,46,47,48].

For the crystallization temperatures, which were only determined in the case of RPD containing PET-based layers, no relevant changes were observed when using the H_2_O_2_ and the steam bag decontamination treatments. Once more, the obtained crystallization temperatures are in the typical ranges found in the literature for PET-based woven fabric materials/products [41,49,50].

However, when employing the NaClO decontamination treatment, a tangible variation was observed at the PET-based layers’ crystallization temperatures from 184.9 °C to 205.6 °C (for the outer PET woven fabric layer), and from 193.4 °C to 209.6 °C (for the inner PET woven fabric layer). This was probably due to the NaClO-assisted extraction/lixiviation of some processing additives that are quite usual at PET-based fabrics (e.g., pigments and flame retardants, as already hypothesized in the TGA results discussion), thus changing free-volume, chain interactions and the crystallization process. Furthermore, it was experimentally observed that the NaClO decontamination treatment was extracting some substances because the remaining liquid solution changed its colour after contact with CM. Surprisingly, the putative removal of these substances did not cause any relevant effects on the melting temperatures of PET-based layers treated with NaClO (as it was also expected to occur). However, and like in the case of WVTR results, the statistical analysis (one-way analysis and Wilcoxon/Kruskal–Wallis test) on melting and crystallization temperature results (Table S6) showed that all the property variations above discussed (Table S5) were not statistically significant (*p*-value > 0.05), which may be attributed to the reduced number of samples analysed, that limits the sensitivity of the statistical analysis.

#### 3.4.6. Mercury Intrusion Porosimetry (MIP)

As mentioned above, the studied decontamination treatments may affect some of the polymeric materials’ morphological properties, namely porosity, average pore diameters, fibre densities and fibre charges of the distinct layers that comprise the RPD, affecting the safety, efficiency and other functional and comfort-related properties of these types of protective devices [25,29,35,41,51]. In this context, mercury intrusion results (Figure S3) showed that after 10 treatment cycles using the H_2_O_2_, NaClO and steam bag treatments, only relatively small variations were observed for the overall porosity (%) and median pore sizes of the original RPD (Table S7). All these RPD comprise several layers with distinct morphologies and therefore results showed that they presented macropores in different porosity ranges: (i) SM masks (0.6 µm; 15 µm; 110 µm); (ii) KN masks (0.5 µm; 22 µm; 65 µm); (iii) CM masks (0.15 µm; 6 µm; 15 µm; 110 µm). For this reason, it was decided to present the median pore size values in Figure S3 and Table S7. In addition, and to the best of our knowledge, hitherto it was not reported in literature any results on porosity and pore sizes determined by mercury intrusion porosimetry for these types of medical devices. Instead, most methods used for these purposes are based on the use of microscopy techniques and image analysis methodologies. For this reason, the results from this work could not be compared with other data reported in the literature.

Finally, the statistical analysis (one-way analysis and Wilcoxon/Kruskal–Wallis test) performed on these results (Table S7) showed that all the observed changes were not statistically significant (*p*-value > 0.05).

#### 3.4.7. Optical and Electronic Microscopy

From the analysis of all SEM micrographs (Figures S8–S10), we could not observe and/or conclude any relevant changes that may have occurred on the surface morphologies of the PP/PET fibres (that comprise the outer layer of studied RPD) and which were originated by the employed decontamination treatments. Nevertheless, the NaClO treatment seemed to introduce some small morphological changes in the PP (for SM and KN masks) and PET (for CM masks) surface fibres. However, these changes may also be artifacts. Optical microscopy images of all the layers of non-processed RPD were also collected and analysed, leading to similar observations.

## 4. Conclusions

In this work, the microbiological effectiveness of three decontamination treatments was assessed on three types of masks (surgical, cloth and KN95), along with their impact on filtration efficiency, air permeability and structural integrity. All three procedures are simple to implement, relatively inexpensive (both in terms of capital costs and operation costs) and can be conducted rapidly. These practical aspects are fundamental for their widespread deployment (scalability) and to leverage an effective response to future pandemics, where a shortage of mask supplies is foreseen and environmental impact from their massive use must be mitigated.

In terms of the microbiology effectiveness of the three procedures, the following results correspond to sterilization (a reduction by 6 orders the magnitude of the bacterial indicator, as established by the ISO standard) or to disinfection (a reduction higher than 3 orders of magnitude and less than 6) can be highlighted:

The nebulized hydrogen peroxide sterilized KN95 and surgical masks. In the case of cloth masks, a reduction of 4 to 5 orders of magnitude of the microbial indicator was obtained using this methodology, which corresponds to disinfection.

The decontamination by immersion in commercial bleach sterilized the surgical masks. In KN95 and cloth masks, a reduction of 5 orders of magnitude was obtained, corresponding to disinfection.

The treatment with steam microwave sterilized KN95 masks. In surgical and cloth masks, disinfection was obtained with 5 and 4 orders of magnitude reduction.

The number of cycles did not have a significant effect on the efficiency of the decontamination processes under analysis.

Concerning the filtration efficiency, air permeability and physicochemical parameters evaluated, this work resulted in several main conclusions:

Cycles of treatments, using any of the three decontamination methods under analysis, did not have a statistically significant impact on filtration efficiency, with the exception of the use of steam bags on KN95 and surgical masks, where the differences were found, even though statistically significant, did not have a major practical impact since the final efficiency was always higher than 97%.

The effects from the treatment cycles were more visible in air permeability. Surgical masks tended to exhibit increasing air permeability with the increase in the number of cycles, whereas cloth masks showed the opposite trend. KN95 mask followed a rather indefinite pattern, but with lower air permeability when compared to the others.

With decontamination treatment cycles, no relevant chemical changes were detected on the surfaces using the ATR-FTIR technique.

Some changes were detected in the contact angle values (and thus wettability) of the distinct layer materials for the KN masks. These changes may be due to some chemical, physical and/or morphological changes induced in layer materials by the decontamination treatments or experimental methods.

The methods WVTR, TGA, MDSC and SEM did not point to substantial changes after 10 cycles of treatments, just showing variations not statistically significant.

Even though the results obtained confirm the potential of the methods, additional studies are required, such as off-gassing experiments to ensure that residual chemicals are not present at potentially harmful levels.

## Figures and Tables

**Table 1:** One-way analysis of filtration efficiency (non-parametric, two-tailed Kruskal–Wallis using ranks). This statistical test was conducted for each combination of RPD (CM, KN, SM) and decontamination treatment (H_2_O_2_, NaClO, Steam Bag), i.e., in a total of 9 distinct cases. The table presents the p-value obtained in each case, where the results obtained across different cycles are compared (control, 5c, 10c); the conditions across the table entries follow the same pattern as in Figure 4. The software used was JMP^®^ PRO ver. 15.0, from SAS Institute Inc.). Statistically significant differences (significance level of 5%) are signalled with an asterisk (*).

Mask/Treatment.	H_2_O_2_	NaClO	Steam Bag
CM	0.7382	0.3220	0.3679
KN	0.5968	0.5750	0.0067 *
SM	0.1194	1.000	0.0024 *

**Table 2:** One-way analysis of air permeability (non-parametric, two-tailed Kruskal–Wallis using ranks). This statistical test was conducted for each combination of RPD (CM, KN, SM) and decontamination treatment (H_2_O_2_, NaClO, Steam Bag), in a total of 9 distinct cases. The table presents the p-value obtained in each such case, where the results obtained across different cycles are compared (control, 5c, 10c); the conditions across the table entries follow the same pattern as in Figure 5. The software used was JMP^®^ PRO ver. 15.0, from SAS Institute Inc.). Statistically significant differences (significance level of 5%) are signalled with an asterisk (*).

Mask/Treatment	H_2_O_2_	NaClO	Steam Bag
CM	0.0014 *	0.5792	0.0033 *
KN	0.0030 *	0.1447	0.0041 *
SM	0.2501	0.0014 *	0.0096 *

## Supplementary Materials

The following supporting information can be downloaded at: https://www.mdpi.com/article/10.3390/ijerph19116567/s1, Figure S1. FTIR-ATR spectra of PP-based and PET-based constituent layers of non-processed RPD. SM, KN and CM correspond to surgical, KN95 and cloth face masks, respectively. In addition: O, I, M, MO and MI indicate the outer layer, the inner layer, the intermediate layer, the intermediate layer closest to the outer layer, and the intermediate layer closest to the inner layer, respectively. Figure S2. WVTR results (from 0-24 hours) for non-processed and processed RPD (10 cycles). SM, KN and CM correspond to surgical, KN95 and cloth face masks, respectively. Experiments were carried out for water vapor flux from the atmosphere to the user (above), and for water vapor flux from the user to the atmosphere (below). In the WVTR units, d stands for 1 day = 24 hours. Figure S3. Porosities (%) and median pore sizes for non-processed and processed RPD (10 cycles) determined by mercury intrusion porosimetry. SM, KN and CM correspond to surgical (left), KN95 (middle) and cloth (right) face masks, respectively. Figure S4. SEM images for surgical masks (SM). Left: bar corresponds to 200 μm. Right: bar corresponds to 10 μm. masks. (A) Control/non-processed; (B) 10 cycles H2O2; (C) 10 cycles NaClO; (D) 10 cycles Steam bag. Figure S5. SEM images for KN95 masks (KN). Left: bar corresponds to 200 μm. Right: bar corresponds to 10 μm. masks. (A) Control/non-processed; (B) 10 cycles H2O2; (C) 10 cycles NaClO; (D) 10 cycles Steam bag. Figure S6. SEM images for cloth masks (CM). Left: bar corresponds to 200 μm. Right: bar corresponds to 10 μm. masks. (a) Control/non-processed; (B) 10 cycles H2O2; (C) 10 cycles NaClO; (D) 10 cycles Steam bag. Table S1. Observed wave number ranges of the bands assigned to poly(propylene) (PP) and poly(ethylene terephthalate) (PET) layers of all non-processed and processed RPD [52,53,54]. Table S2. Static water contact angle results of non-processed and processed RPD layers (10 cycles) and statistical analysis. SM, KN and CM correspond to surgical, KN95 and cloth face masks, respectively. O, I, M, MO and MI indicate the outer layer, the inner layer, the intermediate layer, the intermediate layer closest to the outer layer, and the intermediate layer closest to the inner layer, respectively. Average values and standard deviations are presented. Statistical significances are also indicated (one-way analysis with the Wilcoxon/Kruskal-Wallis test). Table S3 WVTR results of non-processed and processed RPD (10 cycles). SM, KN and CM correspond to surgical, KN95 and cloth masks, respectively. O, I, M, MO and MI indicate the outer layer, the inner layer, the intermediate layer, the intermediate layer closest to the outer layer, and the intermediate layer closest to the inner layer, respectively. Average values and standard deviations are presented. Statistical significances are also indicated (one-way analysis with the Wilcoxon/Kruskal-Wallis test). Table S4. Onset degradation temperatures and ashes/residual masses of non-processed RPD layers. SM, KN and CM correspond to surgical, KN95 and cloth masks, respectively. O, I, M, MO and MI indicate the outer layer, the inner layer, the intermediate layer, the intermediate layer closest to the outer layer, and the intermediate layer closest to the inner layer, respectively. Average values and standard deviations are presented. Table S5. Melting (Tm) and crystallization temperatures (Tc) of non-processed and processed (10 cycles) RPD layers. SM, KN and CM correspond to surgical, KN95 and cloth masks, respectively. O, I, M, MO and MI indicate the outer layer, the inner layer, the intermediate layer, the intermediate layer closest to the outer layer, and the intermediate layer closest to the inner layer, respectively. Average values and standard deviations are presented. Table S6. Statistical analysis on DSC (melting and crystallization temperatures) and mercury intrusion porosimetry (% porosity) results for non-processed and processed RPD (10 cycles). SM, KN and CM correspond to surgical, KN95 and cloth masks, respectively. O, I, M, MO and MI indicate the outer layer, the inner layer, the intermediate layer, the intermediate layer closest to the outer layer, and the intermediate layer closest to the inner layer, respectively. Statistical significances are also indicated (one-way analysis using the Wilcoxon/Kruskal-Wallis test). Table S7. Porosities (%) and median pore diameters for non-processed and processed RPD (10 cycles) determined by mercury intrusion porosimetry. SM, KN and CM correspond to surgical (left), KN95 (middle) and cloth (right) masks, respectively. Average values and standard deviations are presented.
