# Supplementary material for: Evaluation of the Microbiological Effectiveness of Three Accessible Mask Decontamination Methods and Their Impact on Filtration, Air Permeability and Physicochemical Properties
Source: Int J Environ Res Public Health. 2022 May 27;19(11):6567. doi: 10.3390/ijerph19116567 (PMC9180249; doi:10.3390/ijerph19116567)
Supplement: Supplementary file 1 [file ijerph-19-06567-s001.zip › ijerph-1686924-supplementary.pdf]

Supplementary Material

Evaluation of the microbiological effectiveness of three accessible mask decontamination methods and their impact on filtration, air permeability and physicochemical properties

Roberta Lordelo[1], José Rafael S. Botelho[2], Paula V. Morais[1], Hermínio C. de Sousa[2], Rita Branco[1], Ana M. A. Dias[2], Marco S. Reis[2,*]

[1] University of Coimbra, CEMMPRE, Department of Mechanical Engineering, University of Coimbra, 3030-788 Coimbra, Portugal

[2] University of Coimbra, CIEPQPF, Department of Chemical Engineering, Rua Sílvio Lima, Pólo II – Pinhal de Marrocos, 3030-790 Coimbra, Portugal

** Corresponding author: marco@eq.uc.pt*

S1. ATR-FTIR results

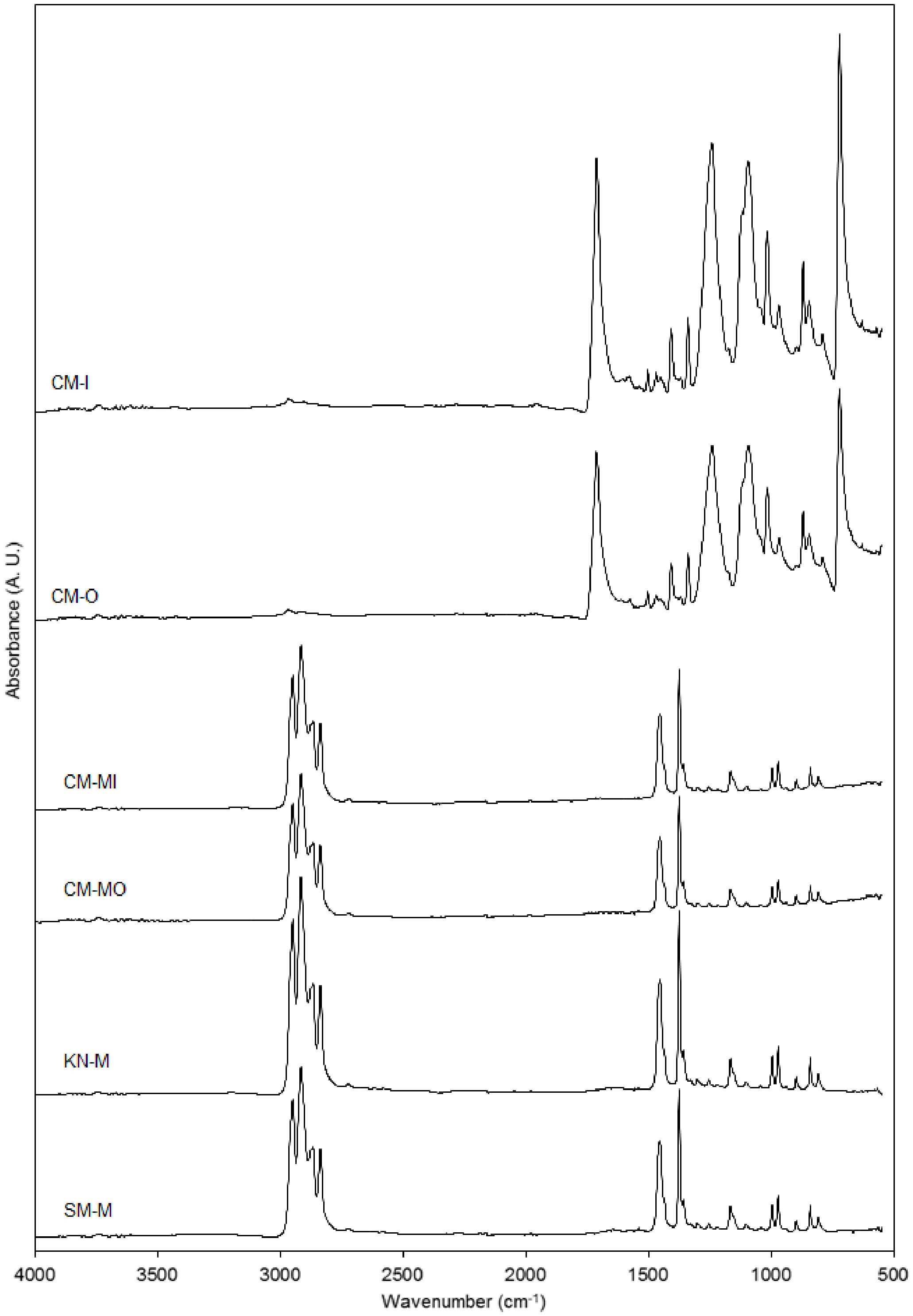

Figure S1. FTIR-ATR spectra of PP-based and PET-based constituent layers of non-processed RPD. SM, KN and CM correspond to surgical, KN95 and cloth face masks, respectively. In addition: O, I, M, MO and MI indicate the outer layer, the inner layer, the intermediate layer, the intermediate layer closest to the outer layer, and the intermediate layer closest to the inner layer, respectively.

Table S1. Observed wave number ranges of the bands assigned to poly(propylene) (PP) and poly(ethylene terephthalate) (PET) layers of all non-processed and processed RPD.

PP		PET	
Peak assignment [a]	Wave number (cm^{-1})	Peak assignment [b]	Wave number (cm^{-1})
C-C	808-810	C-H	722-723
C-H	840-841	C-H	871-872
CH_3<br>C-C	972-973	C-H	968-970
		O=C-O-C	1016-1017
CH_3	996-999	O=C-O-C	1092-1096
C-C<br>C-H<br>CH_3	1166-1169	C-O	1240-1244
		CH_2	1339-1340
		C_6H_6	1408-1409
CH_3	1375-1376	C=O	1711-1714
CH_3	1452-1457		
CH_3	2866-2869		
CH_2	2917		
CH_3	2949-2951		

[a]Fang et al. (2012), ref. [1]
[b]Silva et al. (2012) , ref. [2]

S2. Contact angle measurements

Table S2. Static water contact angle results of non-processed and processed RPD layers (10 cycles) and statistical analysis. SM, KN and CM correspond to surgical, KN95 and cloth face masks, respectively. O, I, M, MO and MI indicate the outer layer, the inner layer, the intermediate layer, the intermediate layer closest to the outer layer, and the intermediate layer closest to the inner layer, respectively. Average values and standard deviations are presented. Statistical significances are also indicated (one-way analysis with the Wilcoxon/Kruskal-Wallis test).

RPD	Layer	Contact angle (º)				*p*-value		
		Control	H_2O_2	NaClO	Steam bag	H_2O_2	NaClO	Steam bag
SM	O	122.6±0.7	124.3±3.4	124.8±1.1	126.4±1.1	0.751	0.143	0.970
	I	126.6±0.9	129.4±1.1	123.9±2.1	127.1±1.4	0.048*	0.205	0.097
	M	126.6±1.3	126.0±1.0	124.2±6.4	137.2±2.5	0.970	0.267	0.003*
KN	O	115.4±0.7	107.3±3.6	111.1±2.1	107.1±2.5	0.108	0.011*	0.028*
	I	140.5±9.9	112.4±2.7	113.1±0.2	122.4±0.9	0.002*	0.003*	0.002*
	M	151.3±1.3	134.8±0.3	139.1±2.1	150.0±6.8	0.005*	0.050*	0.038*
CM	O	132.9±1.1	136.5±2.5	142.4±0.9	146.3±4.1	0.331	0.057	0.005*
	MO	129.2±6.0	129.3±5.5	124.4±1.0	142.5±7.1	0.895	0.386	0.011*
	MI	126.0±1.6	125.3±4.1	133.2±2.2	133.0±0.8	0.154	0.004*	0.013*

*Statistical significance is considered when p-value < 0.05 (Wilcoxon/Kruskal-Wallis test, based on a χ^2 distribution with degrees of freedom equal to the number of levels of contact angle results minus one).

Note: it was not possible to determine water contact angles for the inner layer of CM masks due to the observed rapid water absorption/leakage through PET fabric.

S3. Water vapor transmission rate (WVTR)

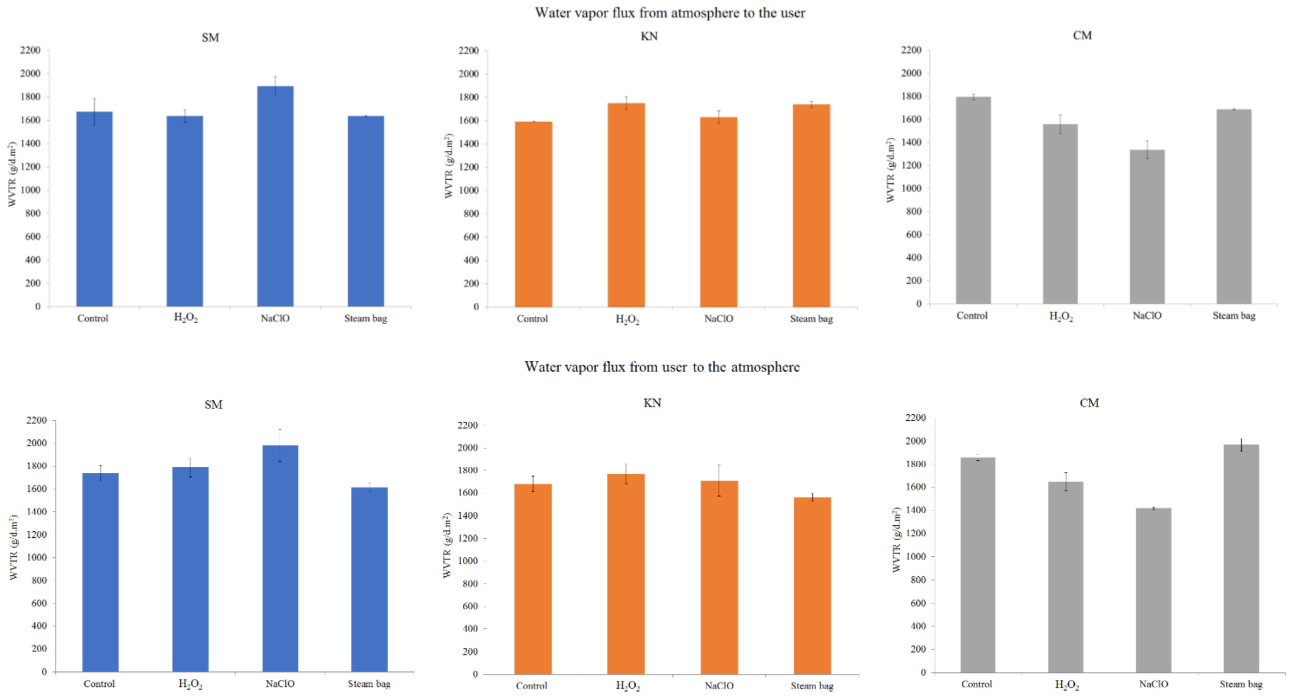

Figure S2. WVTR results (from 0-24 hours) for non-processed and processed RPD (10 cycles). SM, KN and CM correspond to surgical, KN95 and cloth face masks, respectively. Experiments were carried out for water vapor flux from the atmosphere to the user (above), and for water vapor flux from the user to the atmosphere (below). In the WVTR units, d stands for 1 day = 24 hours.

Table S3 WVTR results of non-processed and processed RPD (10 cycles). SM, KN and CM correspond to surgical, KN95 and cloth masks, respectively. O, I, M, MO and MI indicate the outer layer, the inner layer, the intermediate layer, the intermediate layer closest to the outer layer, and the intermediate layer closest to the inner layer, respectively. Average values and standard deviations are presented. Statistical significances are also indicated (one-way analysis with the Wilcoxon/Kruskal-Wallis test).

Flux direction	RPD	Cycle #	WVTR (g/d.m^2)			*p*-value		
			H_2O_2	NaClO	Steam bag	H_2O_2	NaClO	Steam bag
From atmosphere to user	SM	0	1676±108	1676±108	1676±108	0.320	0.123	0.156
		5	1753±55	1831±0	1811±14			
		10	1636±55	1889±83	1818±9			
	KN	0	1597±0	1597±0	1597±0	0.091	0.123	0.095
		5	1596±0	1694±28	1799±21			
		10	1752±55	1635±55	1921±32			
	CM	0	1811±28	1811±28	1811±28	0.165	0.102	0.156
		5	1792±0	1558±165	1839±32			
		10	1577±83	1344±83	1873±5			
From user to atmosphere	SM	0	1739±69	1739±69	1739±69	0.651	0.156	0.156
		5	1694±138	1889±83	1643±26			
		10	1791±88	1983±139	1614±36			
	KN	0	1679±7	1679±7	1679±7	0.180	0.156	0.102
		5	1655±83	1636±55	1590±10			
		10	1770±55	1710±23	1562±18			
	CM	0	1860±27	1860±27	1860±27	0.156	0.180	0.102
		5	1714±110	1422±28	1475±45			
		10	1646±78	1419±11	1969±55			

Statistical significance is considered when p-value < 0.05 (Wilcoxon/Kruskal-Wallis test, based on a $\chi 2$ distribution with degrees of freedom equal to the number of levels of contact angle results minus one).
Note: In WVTR units, d stands for 1 day = 24 hours

S4. Thermogravimetric analysis (TGA)

Table S4. Onset degradation temperatures and ashes/residual masses of non-processed RPD layers. SM, KN and CM correspond to surgical, KN95 and cloth masks, respectively. O, I, M, MO and MI indicate the outer layer, the inner layer, the intermediate layer, the intermediate layer closest to the outer layer, and the intermediate layer closest to the inner layer, respectively. Average values and standard deviations are presented.

RPD	Layer	T_{onset} (ºC)	Residual mass (%)
SM	O	437.9±3.2	0.7±0.3
	I	437.9±0.9	0.4±0.1
	M	438.6±0.2	0.9±0.1
KN	O	438.6±0.8	0.8±0.0
	I	437.8±0.2	1.0±0.2
	M	372.0±0.0	0.1±0.3
CM	O	403.2±6.0	15.7±0.0
	I	403.2±0.2	16.3±1.4
	MO	437.4±0.4	0.4±0.3
	MI	436.6±0.2	0.8±0.1

S5. Modulated Differential scanning calorimetry (MDSC)

Table S5. Melting (T_m) and crystallization temperatures (T_c) of non-processed and processed (10 cycles) RPD layers. SM, KN and CM correspond to surgical, KN95 and cloth masks, respectively. O, I, M, MO and MI indicate the outer layer, the inner layer, the intermediate layer, the intermediate layer closest to the outer layer, and the intermediate layer closest to the inner layer, respectively. Average values and standard deviations are presented.

PP layers				PET layers				
RPD	Cycle	Layer-Treatment	T_m (ºC)	RPD	Cycle	Layer-Treatment	T_m (ºC)	T_c (ºC)
SM	0	O	164.1±0.5	CM	0	O	251.3±1.1	184.9±0.5
	10	O-H_2O_2	163.9±0.2		10	O-H_2O_2	252.3±0.3	184.6±1.1
	10	O-NaClO	162.9±0.2		10	O-NaClO	252.1±0.6	205.6±1.1
	10	O-Steam bag	163.4±0.5		10	O-Steam bag	252.3±0.3	184.5±1.4
	0	I	163.1±1.0		0	I	251.9±0.9	193.4±1.1
	10	I-H_2O_2	162.9±0.2		10	I-H_2O_2	251.9±0.9	190.1±1.6
	10	I-NaClO	163.9±0.7		10	I-NaClO	251.6±1.2	209.6±4.3
	10	I-Steam bag	164.6±0.7		10	I-Steam bag	252.7±0.2	190.9±0.5
	0	M	164.3±0.2					
	10	M-H_2O_2	164.3±0.1					
	10	M-NaClO	164.5±0.4					
	10	M-Steam bag	163.8±0.9					
KN	0	O	167.3±0.5					
	10	O-H_2O_2	166.9±0.8					
	10	O-NaClO	166.7±0.3					
	10	O-Steam bag	167.4±0.1					
	0	I	166.4±0.6					
	10	I-H_2O_2	166.7±1.1					
	10	I-NaClO	166.3±0.2					
	10	I-Steam bag	166.6±0.2					
	0	M	164.1±0.0					
	10	M-H_2O_2	164.5±0.7					
	10	M-NaClO	164.5±0.2					
	10	M-Steam bag	165.3±0.9					
CM	0	MO	163.1±0.5					
	10	MO-H_2O_2	162.7±0.5					
	10	MO-NaClO	163.8±0.7					
	10	MO-Steam bag	162.4±0.9					
	0	MI	162.5±0.7					
	10	MI-H_2O_2	162.4±0.7					
	10	MI-NaClO	163.9±0.7					
	10	MI-Steam bag	162.6±0.9					

Table S6. Statistical analysis on DSC (melting and crystallization temperatures) and mercury intrusion porosimetry (% porosity) results for non-processed and processed RPD (10 cycles). SM, KN and CM correspond to surgical, KN95 and cloth masks, respectively. O, I, M, MO and MI indicate the outer layer, the inner layer, the intermediate layer, the intermediate layer closest to the outer layer, and the intermediate layer closest to the inner layer, respectively. Statistical significances are also indicated (one-way analysis using the Wilcoxon/Kruskal-Wallis test).

Technique/Property	RPD	Layer	*p*-value		
			**H_2O_2**	**NaClO**	**Steam bag**
**DSC**					
T_m	SM	O	0.683	0.121	0.221
		I	1.000	0.439	0.121
		M	0.683	0.439	0.683
	KN	O	0.439	0.121	1.000
		I	0.683	1.000	0.683
		M	0.683	0.121	0.121
	CM	O	0.221	0.439	0.221
		MO	0.439	0.439	0.439
		MI	0.683	0.121	0.683
		I	0.439	0.683	0.1213
T_c	CM	O	1.000	0.121	1.000
		I	0.121	0.121	0.121
**Mercury intrusion porosimetry**					
Porosity (%)	SM	Full device	0.439	0.439	1.000
	KN	Full device	1.000	0.121	1.000
	CM	Full device	0.439	0.121	1.000

Statistical significance is considered when p-value < 0.05 (Wilcoxon/Kruskal-Wallis test, based on a $\chi 2$ distribution with degrees of freedom equal to the number of levels of contact angle results minus one).

S6. Mercury intrusion porosimetry (MIP)

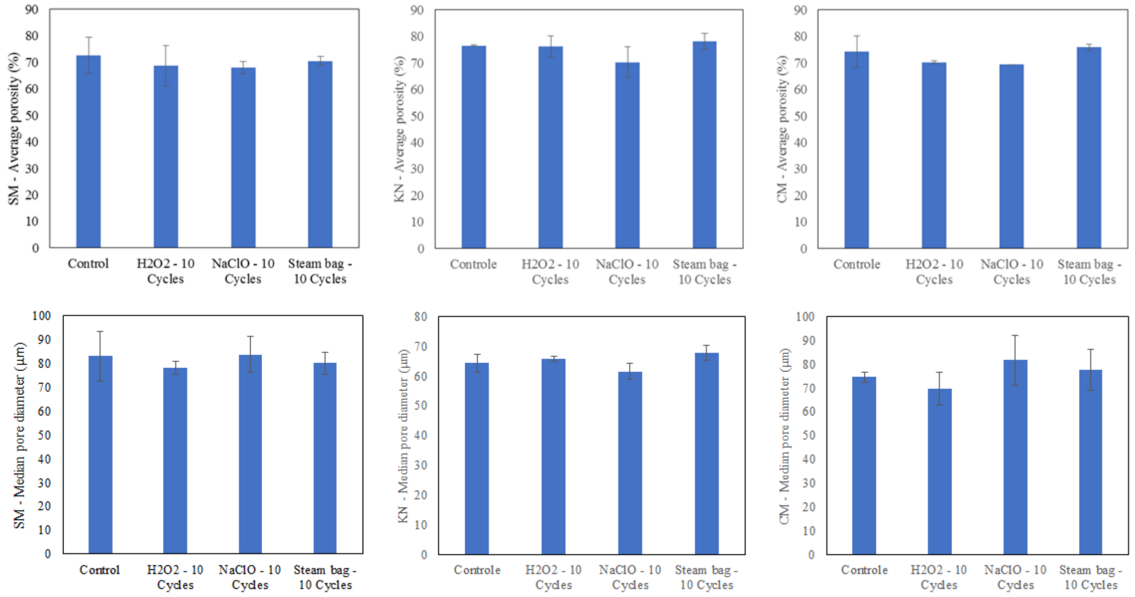

Figure S3. Porosities (%) and median pore sizes for non-processed and processed RPD (10 cycles) determined by mercury intrusion porosimetry. SM, KN and CM correspond to surgical (left), KN95 (middle) and cloth (right) face masks, respectively.

Table S7. Porosities (%) and median pore diameters for non-processed and processed RPD (10 cycles) determined by mercury intrusion porosimetry. SM, KN and CM correspond to surgical (left), KN95 (middle) and cloth (right) masks, respectively. Average values and standard deviations are presented.

RPD	Cycle	Treatment	Porosity (%)	Median pore size (µm)
SM	0	Control	73±7	83±11
	10	H_2O_2	69±8	78±3
	10	NaClO	68±2	84±8
	10	Steam bag	70±2	80±5
KN	0	Control	77±0	64±3
	10	H_2O_2	76±4	66±1
	10	NaClO	70±6	62±3
	10	Steam bag	78±3	68±2
CM	0	Control	74±6	75±2
	10	H_2O_2	70±0	70±7
	10	NaClO	69±0	82±10
	10	Steam bag	76±1	78±9

S7. Optical and Electronic Microscopy

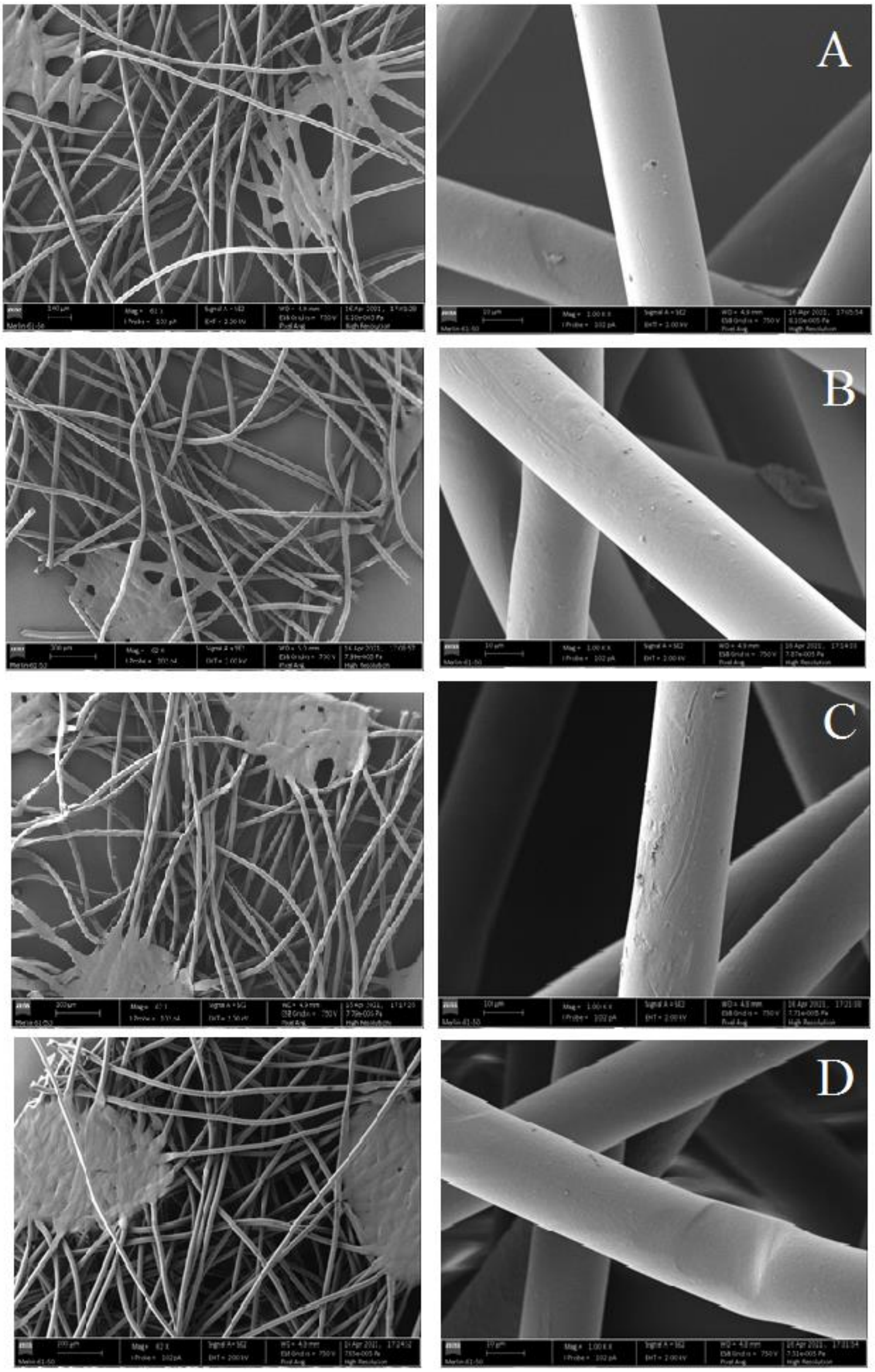

Figure S4. SEM images for surgical masks (SM). Left: bar corresponds to 200 µm. Right: bar corresponds to 10 µm. masks. (A) Control/non-processed; (B) 10 cycles H_2O_2; (C) 10 cycles NaClO; (D) 10 cycles Steam bag.

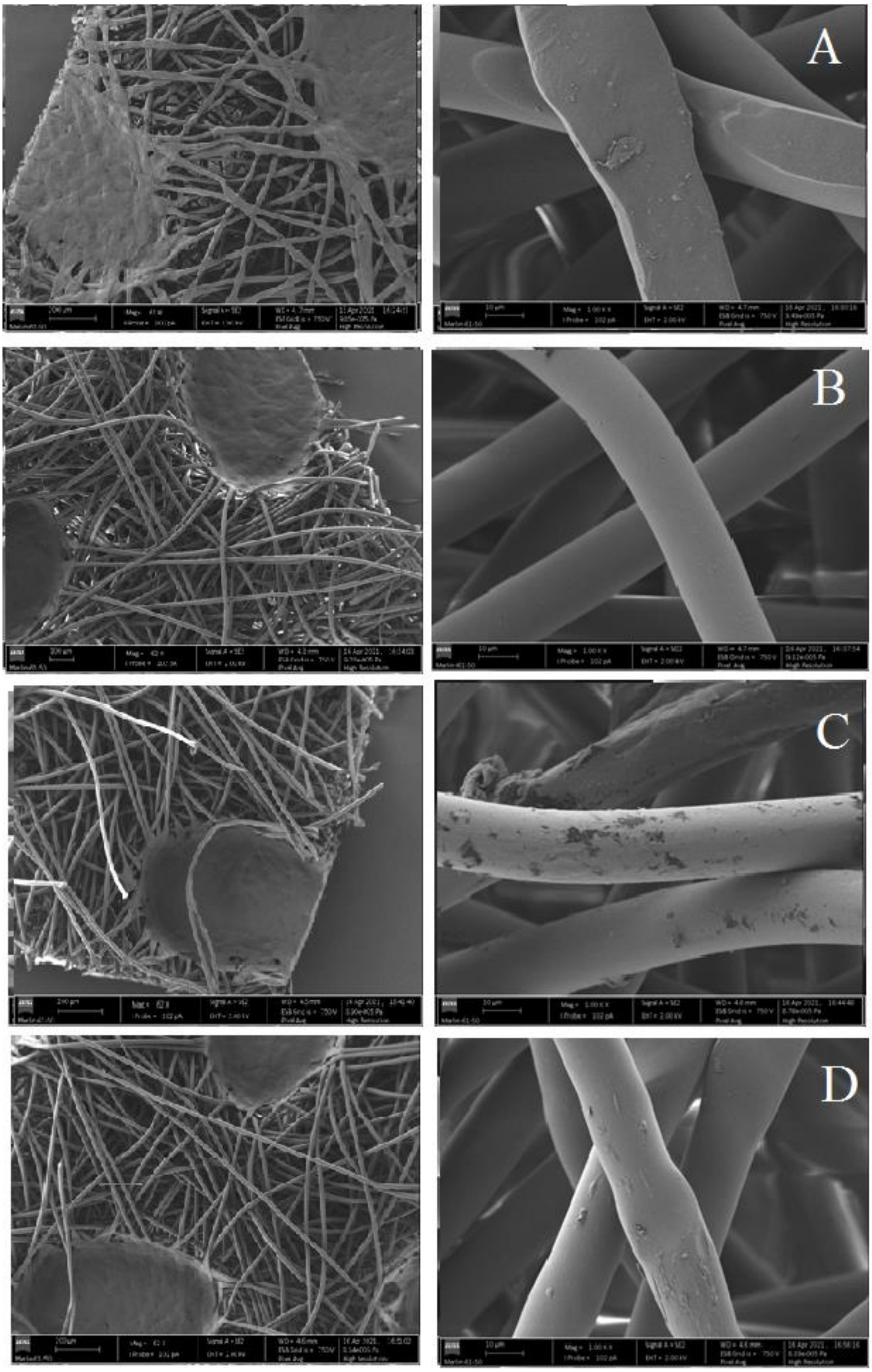

Figure S5. SEM images for KN95 masks (KN). Left: bar corresponds to 200 µm. Right: bar corresponds to 10 µm. masks. (A) Control/non-processed; (B) 10 cycles H_2O_2; (C) 10 cycles NaClO; (D) 10 cycles Steam bag.

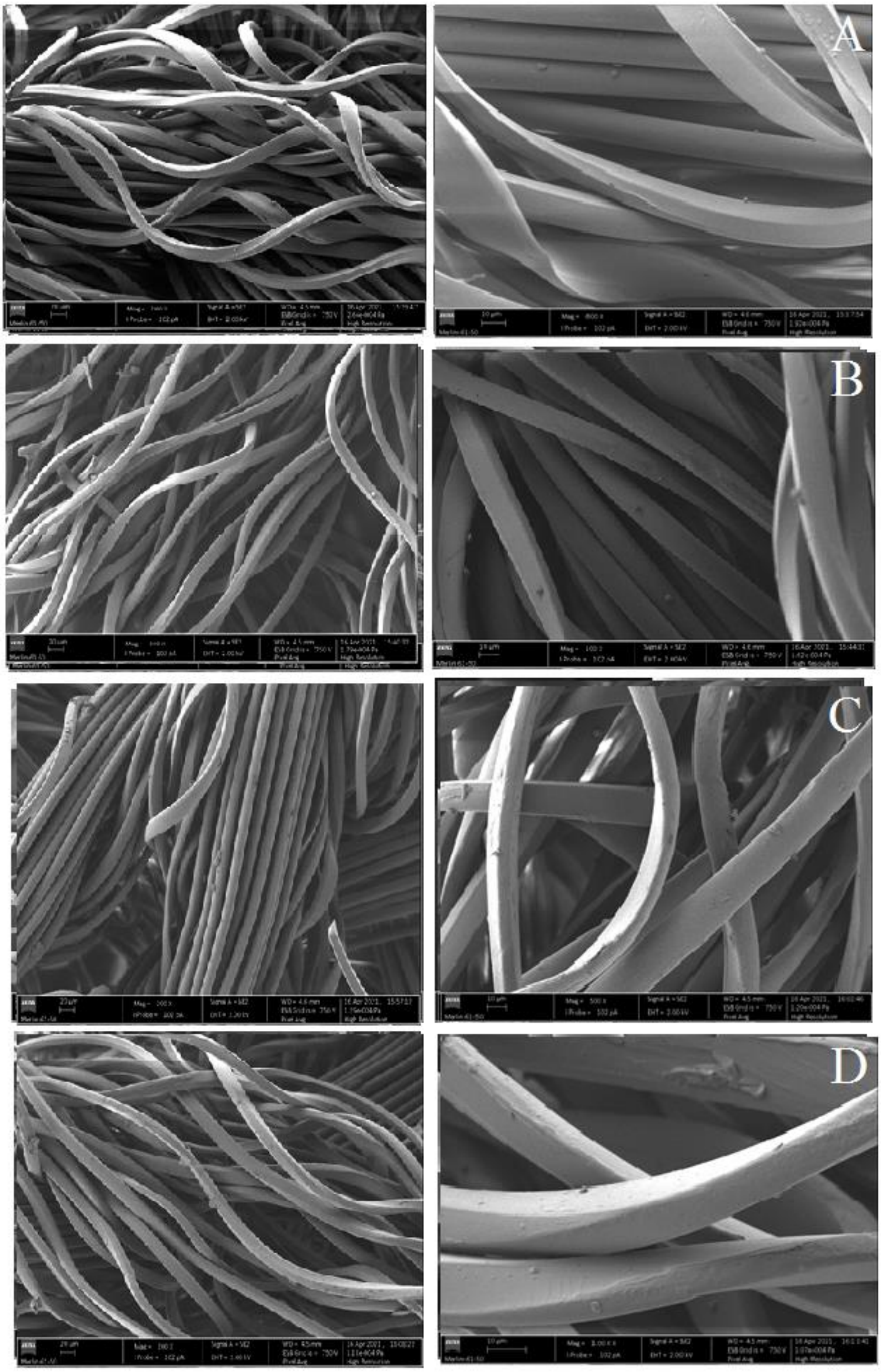

Figure S6. SEM images for cloth masks (CM). Left: bar corresponds to 200 µm. Right: bar corresponds to 10 µm. masks. (a) Control/non-processed; (B) 10 cycles H_2O_2; (C) 10 cycles NaClO; (D) 10 cycles Steam bag.

References

1. Fang, J.; Zhang, L.; Sutton, D.; Wang, X.; Lin, T. Needleless melt-electrospinning of polypropylene nanofibres. *Journal of Nanomaterials* **2012**, *2012*, 382639.

2. da Silva, R.C.L.; Alves, C.; Nascimento, J.H.; Neves, J.R.O.; Teixeira, V. Surface modification of polyester fabric by non-thermal plasma treatment. *Journal of Physics: Conference Series* **2012**, *406*, 012017.

3. Elnagar, K.; Abou Elmaaty, T.; Raouf, S. Dyeing of polyester and polyamide synthetic fabrics with natural dyes using ecofriendly technique. *Journal of Textiles* **2014**, *2014*, 363079.
